# Ultra-broadband diffractive imaging with unknown probe spectrum

**DOI:** 10.1038/s41377-024-01581-4

**Published:** 2024-08-26

**Authors:** Chuangchuang Chen, Honggang Gu, Shiyuan Liu

**Affiliations:** 1 State Key Laboratory of Intelligent Manufacturing Equipment and Technology, Huazhong University of Science and Technology, Wuhan, Hubei 430074 China; 2 Optics Valley Laboratory, Wuhan, Hubei 430074 China

**Keywords:** Imaging and sensing, Optical metrology

## Abstract

Strict requirement of a coherent spectrum in coherent diffractive imaging (CDI) architectures poses a significant obstacle to achieving efficient photon utilization across the full spectrum. To date, nearly all broadband computational imaging experiments have relied on accurate spectroscopic measurements, as broad spectra are incompatible with conventional CDI systems. This paper presents an advanced approach to broaden the scope of CDI to ultra-broadband illumination with unknown probe spectrum, effectively addresses the key challenges encountered by existing state-of-the-art broadband diffractive imaging frameworks. This advancement eliminates the necessity for *prior* knowledge of probe spectrum and relaxes constraints on non-dispersive samples, resulting in a significant extension in spectral bandwidth, achieving a nearly fourfold improvement in bandlimit compared to the existing benchmark. Our method not only monochromatizes a broadband diffraction pattern from unknown illumination spectrum, but also determines the compressive sampled profile of spectrum of the diffracted radiation. This superiority is experimentally validated using both CDI and ptychography techniques on an ultra-broadband supercontinuum with relative bandwidth exceeding 40%, revealing a significantly enhanced coherence and improved reconstruction with high fidelity under ultra-broadband illumination.

## Abstract (web summary)

This method monochromatizes broadband diffraction patterns from unknown ultra-broadband illumination while simultaneously determining the diffracted radiation’s spectral features in an ultra-broadband imaging system.

## Graphical abstract

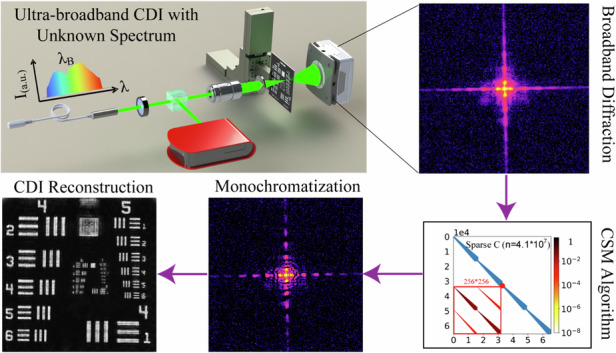

## Introduction

Coherent diffraction imaging (CDI) is an elegant lensfree computational imaging technology to high-resolution imaging fields^1–3^ The core issue in CDI is the retrieval of phase information from the captured diffraction frame. Various frameworks have been developed to recover the missing phase in CDI over the past decades^4–7^, and great improvements based on CDI have been promoted alternatively. For instance, Fourier holography^8,9^ directly captures the phase distribution by interference with a separate reference wave. Ptychography^10–13^ records multiple overlapped diffraction patterns to retrieve a wide-field image. Fourier ptychography^14,15^ stitches together a number of variably illuminated, low-resolution intensity images in Fourier space to produce a high-resolution image.

Full coherence of illumination is generally assumed in CDI, driven by the inherent chromaticity of diffractive optics that the diffracted angle from any microstructure only depends on its wavelength. A diffraction pattern for a varying spectrum channel undergoes a spatial scaling towards the corresponding wavelength. Thus, the extension in spectrum results in diffraction aliasing, preventing CDI from correct convergence^16^. Practically, a necessary coherence filtering is commonly processed to select a quasi-monochromatic radiation from the source spectrum CDI applications^17–19^, which brings a significant barrier to the photon efficiency of full spectrum. Novel strategies are required to overcome the trade-off between radiation bandwidth and convergence for broadband imaging.

The first utilization of broadband CDI (BCDI) introduced by Fienup in 1999 has opened a new window to characterize a broadband radiation from multi-wavelength mapping with insufficient number of wavelengths^20^. Since then, researchers have conducted further studies around this issue for decades. Imaging with a partially coherent wavefront can be cast as a blind deconvolution problem with several discrete wavelength channels, where the mixed states of decoherence can be deconvolved by advanced reconstruction algorithms^21^, such as ptychographic information multiplexing (PIM)^22–24^ and multi-wavelength techniques^25–27^. An alternative approach, called polyCDI, extends the phase retrieval algorithm and demonstrates convergence with a pre-measured spectrum of 3% bandwidth^28,29^. Recent developments in broadband ptychography enable the imaging of extended objects using a freely referenced spectrum^30–33^. However, these approaches involve complex iterative computations across the dense wavelength channels of the full spectrum. The convergence is highly sensitive to the spectral bandwidth, typically limited to 5% bandlimit. BCDI techniques, such as mono CDI^34^ and two-pulse imaging^35^, achieve an extended bandwidth of up to 11% experimentally but have challenges. Mono CDI relies on accurately pre-measured spectrum, while two-pulse imaging depends on complex opto-mechanical Fourier spectrometer designs. Overall, these solutions face formidable challenges, including intricate iterative computations across dense wavelength channels, the need for accurate spectrum measurement, strict constraints for non-dispersive specimens over the full spectrum, and convergence within the bandlimit for validity. These challenges impede progress in ultra-wide spectrum broadband diffractive imaging.

In a recent development, we introduced a ultra-streamlined diffraction-based computational spectrometer based on the coherent mode decomposition from broadband diffraction measurement^36^. The implementation of this computational spectrometer within the context of broadband computational imaging marks a significant advancement in recovering the compressive sampled profile of spectrum (CSS) of the imaging system. Drawing inspiration from the mono CDI framework, we further propose an advancement to broaden the scope of CDI to ultra-broadband illumination with unknown probe spectrum, termed ultra-broadband diffractive imaging (UDI). UDI, for the first time, eliminates the need for *prior* knowledge of probe spectrum and relaxes constraints on non-dispersive samples, achieving significant enhancement in photon efficiency for ultra-broadband computational imaging, effectively addresses the key challenges encountered by existing state-of-the-art broadband diffractive imaging frameworks. This innovation not only reconstructs the CSS of the diffracted radiation, but also achieves a coherence-enhanced and superfast-solving monochromatization (CSM) of the captured broadband pattern with high efficiency. Crucially, the monochromatization in UDI is exclusively reliant on the recovered CSS, circumventing the need for spectrum measurement and overcoming limitations imposed by the constraint of spectrally non-dispersive specimens. The superiority of UDI is experimentally confirmed using both CDI and ptychography from an ultra-broadband spectrum with relative bandwidth exceeding 40%, revealing a precise spectrum measurement and a super-fast and robust monochromatization convergence with no need for *prior* spectral knowledge. This is particularly advantageous for in-line broadband imaging applications where efficiency and speed are crucial. To the best of our knowledge, this is the first demonstration of an ultra-broadband CDI comprising an ultra-simplified design, while eliminating the constraint of non-dispersion for the specimen or the need for accurate knowledge of probe spectrum, providing a successful ultra-broadband CDI with a significant improvement in photon utilization efficiency and remarkable enhancement in coherence across the entire spectrum. The superiority of the proposed UDI is compared in Table 1.

**Table 1:** Comparison among State-of-the-art broadband diffractive imaging methods

Method	Spectrum knowledge	Non-dispersive object assumption	Bandwidth (FWHM)	Computational complexity
Mixed-State^21^	No	No	Several harmonics	Moderate
PIM^22–24^	Yes	Yes	Several harmonics	Moderate
Multiwavelength^25–27^	Yes	Yes	Several harmonics	Moderate
Poly CDI^28^	Yes	Yes	3%	High
Mono CDI^34,42^	Yes	Yes	11%	Low
SPIRE^31^	No	No	28%	Extremely High
BBSSP^30^	No	No	5.6%	Extremely High
*This work*	*No*	*No*	41%	*Extremely low*

## Results

### UDI operation

**Fig. 1:**
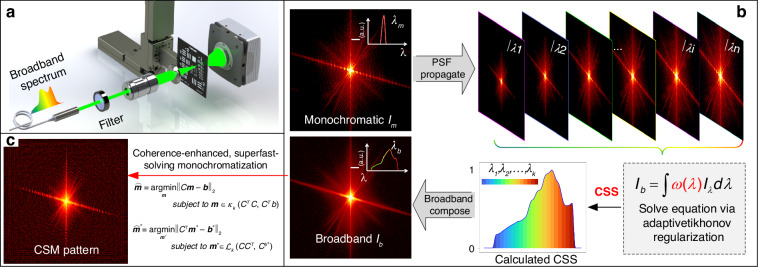
Principle of UDI operation. **a** Geometry of UDI operation. A spectral filter is placed to modulate a quasi-monochromatic or broadband illumination the from a supercontinuum source. **b** PSF mapping from a monochromatic diffraction at wavelength *λ*_*m*_. A broadband diffraction *I*_*b*_ captured in-situ can be thought of as a superposition of PSFs of *I*_*m*_ at different wavelengths over full spectrum, each multiplied by its corresponding power spectrum weighting *ω*(*λ*). The CSS is reconstructed via adaptive Tikhonov regularization. **c** Monochromatization procedure consists in the inversion of the ill-posed matrix function *I*_*b*_ = C*I*_*m*_ to retrieve the CSM pattern from the solved CSS

As the schematic principle demonstrated in Fig. 1, since a broadband pattern *I*_*b*_ diffracted by a microstructure can be interpreted as a linear superposition of multiple discrete channels of monochromatic diffraction patterns in the source spectrum^28^, each individual-wavelength diffraction profile *I*_*λ*_ at channel *λ* can be characterized by a snapshot of a quasi-monochromatic diffraction measurement *I*_*m*_ at *λ*_*m*_ by utilizing the spatial-spectral point-spread function (PSF) mapping scheme with a scaling factor *λ*_*i*_/*λ*_*m*_, as shown in Fig. 1b schematically. The broadband pattern can be treated as the incoherent sum of all spectrum components, given by the sum of PSFs weighted by the power spectrum *ω*(*λ*) of the scattered light, rewritten to a matrix form in simplicity:

1 $${I}_{b}=\mathop{\sum }\limits_{{\rm{i}}=1}^{n}\omega ({\lambda }_{i}){[PSF({\lambda }_{i})]}^{2}$$

where the *PSF*(*λ*_*i*_) is a spectrum propagation function from a reference diffraction field $\sqrt{{I}_{m}}$ (details in Supplementary S1). A broadband measurement *I*_*b*_ with M*N pixels can be represented as the integral of *ω*(*λ*)[*PSF*(*λ*)]^2^ over the wavelength range, including of M*N multi-linear equations with *n* parameters. Practically, it is usually impossible to solve *ω*(*λ*) by ordinary noniterative methods due to its ill-posed nature. To tackle such instabilities, we perform an improved residual norm minimization tactic applied with a weighting regularization factor, known as Tikhonov regularization^37–39^ to solve Eq. (1). As a result, an optimal CSS estimate of the original spectrum *ω*(*λ*) is extracted (detailed in Methods and the supplementary information in ref. ^36^).

Importantly, since *ω*(*λ*) represents neither the probe spectrum *P*(*λ*) nor the diffracted radiation, but the final corrected spectrum for the sample’s spectral transmissivity function *T*(*λ*) and the quantum efficiency of detector *QE*(*λ*), *ω*(*λ*)= *P*(*λ*) *T*(*λ*) *QE*(*λ*). Thus, the CSS represents the principal component of the final corrected power spectrum, which considers the light-matter interaction between the broadband diffractive radiation through the sample and the diffraction photons read out by the detector over the full spectrum. Thus, there is no need to correct the spectrum for the detector response or make the strong constraint of non-dispersive specimen over the entire spectrum for BCDI. Practically, the CSS is calculated just once and can be applied to various non-dispersive samples. In situations involving dispersive objects, the objects spectral transmissivity can also be obtained from the CSS matrix.

As outlined in mono CDI, the retrieval of the monochromatic pattern can be further reduced to a linear algebra problem, rewritten to a matrix form in simplicity

2 $${C}^{T}{\boldsymbol{b}}={C}^{T}C{\boldsymbol{m}}$$

where ***m*** stands for the vector of the monochromatic pattern, ***b*** represents the broadband pattern, and C can be regarded as containing the spectrally dependent PSFs over the calculated CSS. Here, we adopt a specific expression to calculate C in one dimension (detailed in Supplementary S2). Note that C is fully determined by the calculated CSS and the dimension of the measured broadband pattern (Fig. 1c). For a 2D diffraction pattern with 512 × 512 pixels, C is a 4D matrix with 256^4^ values. Thanks to the sparsity of the CSS, C is also sparse, with only a few percent of non-zero values. Crucially, once the CSS is computed within the framework of a BCDI configuration, matrix C attains a unique determination, rendering it entirely independent of the specific broadband patterns employed in BCDI. Given its nature as a matrix characterized by high ill-posedness, sparsity, symmetry, and positive definiteness, the direct application of conventional noniterative methods to solve Eq. (2) is typically deemed impractical. Nonetheless, the sparsity and positive definiteness attributes of matrix C render it notably amenable to iterative solutions, particularly through the utilization of the Conjugate Gradient-based descent algorithms. In this work, we employ a normalized BiCGStab^40^ for UDI (see Section Methods and Supplementary S3). The optimized monochromatization quickly converges within the initial iterations. The numerical implementation of BiCGStab includes two additional constraints: non-negativity of monochromatization and a support constraint on the initial guess of ***m*** set to the broadband measurement ***b***. These constraints prevent overfitting and enhance the regularization effectiveness of the method.

### Ptychographic UDI experiments

**Fig. 2:**
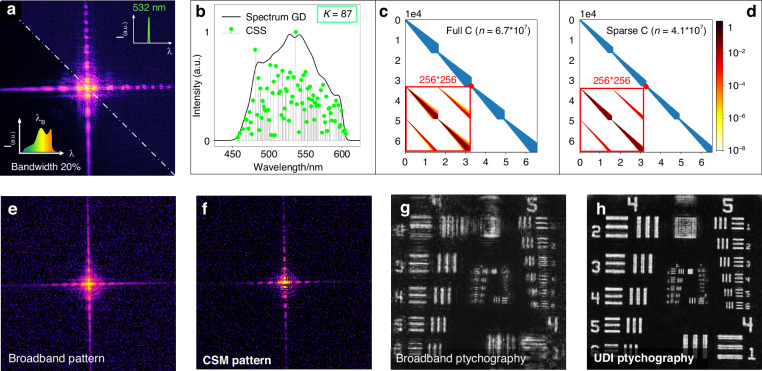
UDI ptychography at 20% bandwidth. **a** The pre-captured quasi-monochromatic diffraction pattern at 532 nm (up right) and the corresponding broadband pattern captured in-situ (bottom left) to calculate the CSS. **b** The broadband source spectrum (black curve) and the recovered CSS consisting of 87 sparse spectral profiles (green scatters). **c**. Matrix C generated from the spectrum measurement in **a**. **d** A comparison of the sparse matrix C obtained from the CSS in **b**. **e** A frame of the broadband pattern. **f** The corresponding CSM pattern recovered from the broadband data in **e**. **g** and **h** depict the reconstructed images from the broadband ptychography and the proposed UDI ptychography, respectively, after 600 iterations of mPIE

We firstly present a broadband ptychography conducted with a bandwidth of 20% to illustrate the performance of UDI (experimental set-up detailed in Supplementary S4). Initially, we conducted a capture of coherent diffraction at 532 nm with a 3 nm full width at half maximum (FWHM) and broadband diffraction in-situ at any identical position of the USAF target (Fig. 2a). The CSS was then extracted with 87 sparse spectrum channels (green scatters in Fig. 2b). Following the extraction of the CSS, the sparse matrix C is subsequently computed (Fig. 2d), allowing us to monochromatize the broadband measurements. Additionally, we also computed the matrix C from the dense spectrum measurement (Fig. 2c). It is evident that the CSS-derived matrix C contains only 4.1 × 10^7 non-zero values sparsely, compared to the full spectrum-derived matrix C with 6.7 × 10^7 non-zero values. This sparsity is a result of the low-energy trend of spectral leakage in CSS, which causes the CSS-derived matrix C to exhibit a sparser characteristic. It should be mentioned that the matrix C is just performed only once per spectrum and can be used for varying non-dispersive samples.

A comparison of the broadband measurement and the corresponding narrowband pattern captured in-situ reveals that the use of broadband illumination introduces a noticeable decoherence (Fig. 2e). Afterwards, we applied the CSM approach to monochromatize the broadband measurements. The enhancement of coherence between the broadband pattern (Fig. 2e) and the CSM result (Fig. 2f) is readily apparent, revealing that the CSM in UDI is remarkably efficient with only single iteration of monochromatization calculation. Utilizing this efficiency, we monochromatized all the broadband measurements, resulting in monochromatization with notably enhanced coherence and superfast convergence. Subsequently, a comparison between the broadband ptychography and the proposed UDI ptychography were performed using 600 iterations of the mPIE^11^, respectively. Seeing that all elements in group 6 of the USAF target reconstructed from the UDI results (Fig. 2h) exhibits clear and high-fidelity resolution compared to that obtained from the original broadband datasets (Fig. 2g). This excellent agreement in monochromatization and the high quality of the ptychographic result serve as strong validation for the effectiveness of our UDI approach in the realm of broadband ptychography. Besides, a revised evaluation matric is proposed as an improved evolution function to monitor the evolution for the broadband ptychography, as detailed in Supplementary S5.

**Fig. 3:**
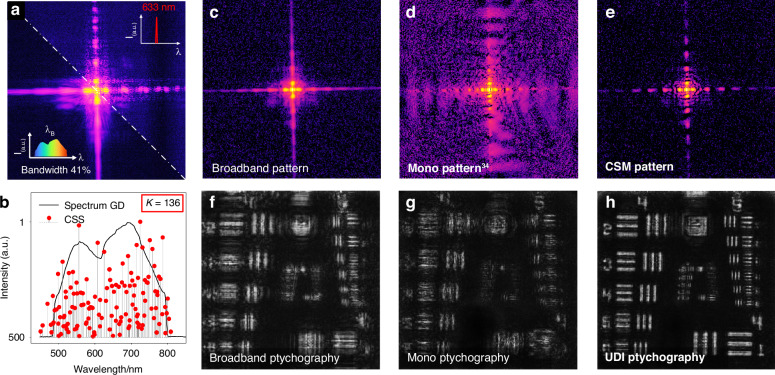
UDI ptychography at 41% bandwidth. **a** Same as Fig. 2a but narrowband filter at 633 nm and broadband filter with 41% FWHM. **b** The calculated CSS consisting of 136 sparse spectral profiles (red scatters). **c**–**e** A frame of the measured broadband pattern, the corresponding monochromatized pattern via published mono CDI framework^34^ (optimum 25 iterations), and the proposed CSM pattern, respectively. **f**–**h** compare the reconstructed results from the broadband ptychography, mono ptychography, and UDI ptychography, respectively

We further extended the bandwidth of the source spectrum to 41% FWHM, and repeated the UDI ptychography experiment. The CSS can still be precisely computed (green scatters in Fig. 3b) from the diffraction signals (Fig. 3a). It is noteworthy that the spectral extension of the light source results in significant diffraction aliasing (Fig. 3c), ultimately leading to the failure of broadband ptychography convergence (Fig. 3f). The existing mono CDI^34^ fails to converge under the ultra-wide spectrum (Figs. 3d, 3g). However, UDI still effectively addresses the decoherence issue arising from the ultra-wide spectral radiation. The CSM pattern in Fig. 3e exhibits significantly enhanced coherence compared to the broadband measurement in Fig. 3c. Moreover, the UDI ptychography result in Fig. 3h also showcases an enhancement in reconstruction fidelity for ultra-broadband diffractions. Note that the reconstruction in Fig. 3h shows an evident decrease in resolution compared to that with a bandwidth of 20% FWHM in Fig. 2h. This decrease is due to severe aliasing of high-frequency diffraction information in the ultra-broadband diffraction signal, which prevents the accurate coherent recovery of high-frequency diffraction features during monochromatization. Ultimately, this hinders the further enhancement of resolution under ultra-wide spectral illumination. We conducted a detailed comparison of the monochromatization evolution between the CSM algorithm and the conjugate gradient least squares (CGLS) algorithm used in mono CDI^41^, as elaborated in Supplementary S6.

### BCDI experiments

**Fig. 4:**
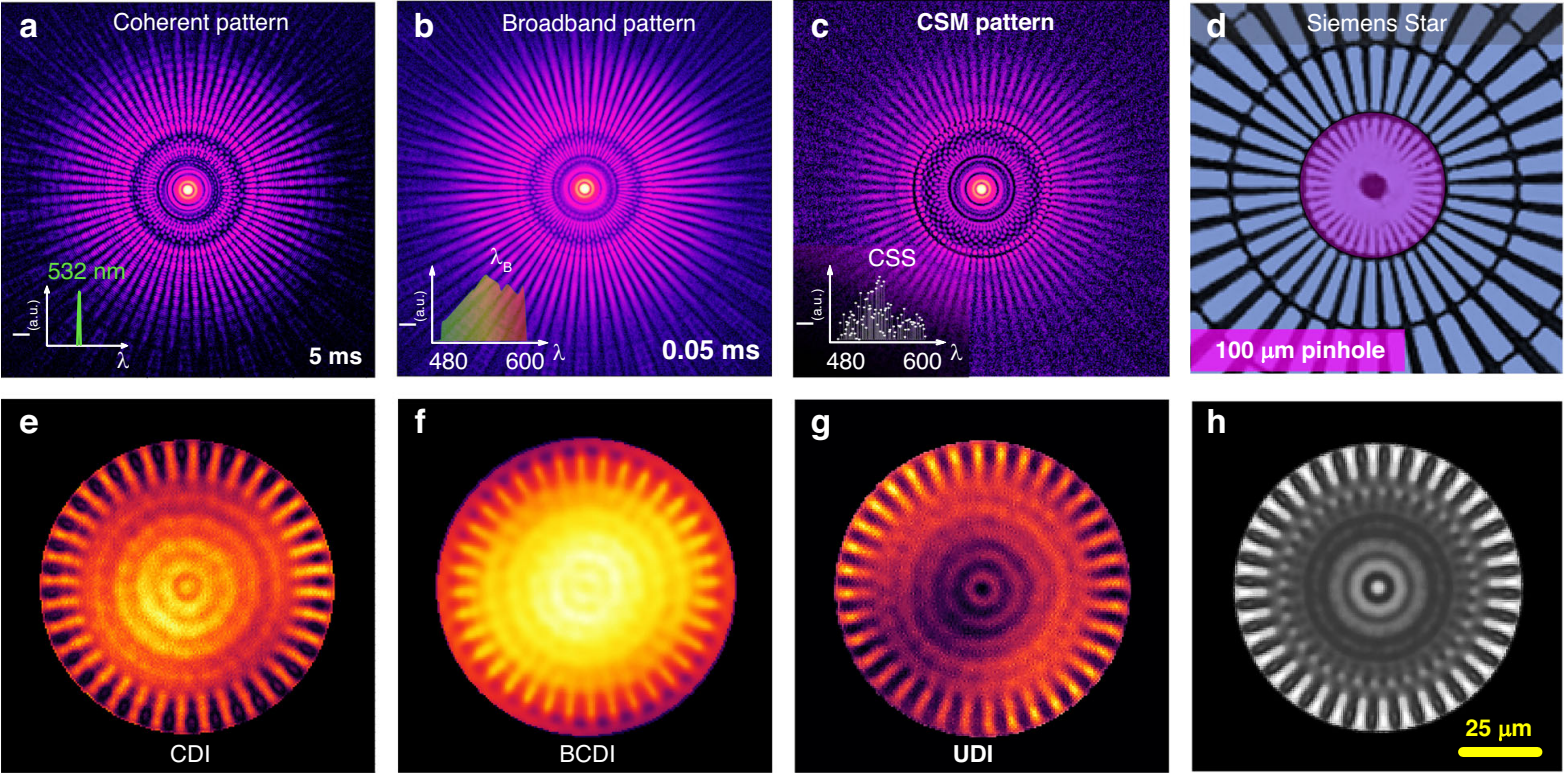
Broadband diffractive imaging at 20% bandwidth **a** The pre-captured coherent pattern at 532 nm with 5 ms exposure time. **b** The corresponding broadband pattern captured in-situ with 20% bandwidth (spectrum ranging from 480 nm to 600 nm) with only 0.05 ms exposure time. **c** The CSM pattern recovered from the broadband data in **b**. **d** A photograph of the Siemens star target, a micro pinhole with100 μm in diameter is employed to intercept the incoming light illumination, resulting in a circular planar wave approximately 100 μm that is incident on the Siemens star. **e**–**g** depict the reconstructed patterns obtained from the CDI in **a**, BCDI in **b**, and the proposed UDI in **c**, respectively, after 500 iterations of RAAR. **h** shows an average image of ten trails of UDI reconstructions

Additionally, a BCDI application is also showcased in Fig. 4, where the experimental setup resembles that of broadband ptychography. Mentioning that all CDI procedures were performed using the RAAR algorithm with 500 iterations. We first captured a shot of coherent diffraction pattern at 532 nm using a bandpass filter with a 3 nm FWHM (Fig. 4a). The corresponding CDI result is shown in Fig. 4e. To assess the impact of decoherence, we conducted an in-situ acquisition of a broadband pattern with a bandwidth of 20% FWHM, spanning from 480 nm to 600 nm (Fig. 4b). The decoherence nature of the broadband pattern ultimately resulted in a convergence failure in BCDI (Fig. 4f). In comparison, the CSM pattern (Fig. 4c) exhibits a notably enhanced coherence. Simultaneously, the UDI method recovers the probe spectrum, as illustrated by the CSS plotted in Fig. 4c. Furthermore, the UDI reconstructions (Fig. 4g, h) reveal a remarkable improvement in the fidelity of the reconstructions. Importantly, the UDI approach seamlessly combines the inherent advantage of high coherence in monochromatic diffraction with the high photon utilization efficiency offered by full spectrum illumination. Through the enhancement of coherence, the photon utilization efficiency is boosted by two orders of magnitude, leading to a significant reduction in detector acquisition time. Specifically, for broadband illumination, the detector acquisition time is reduced to only 0.05 ms compared to 5 ms for coherent illumination. This improvement in both efficiency and coherence contributes significantly to the overall superiority of the UDI method.

### Broadband diffractive imaging with spectrally dispersive specimen

We are further considering a more general case of broadband diffractive imaging, where the specimen is spectrally dispersive. Most of the current state-of-the-art research on BCDI relies heavily on the strong assumption that the specimen should be non-dispersive over the spectrum^26,28,34,42–44^. This assumption severely restricts the applicability of broadband imaging, particularly in the extreme ultraviolet (EUV) and soft X-ray spectral ranges where the material’s absorption edge effect is more prominent^8,45^. The proposed UDI approach effectively addresses these limitations, allowing for the extraction of both the probe spectrum as well as the specimen’s dispersiveness using the CSS, enabling the application of BCDI to spectrally dispersive specimens with ease.

**Fig. 5:**
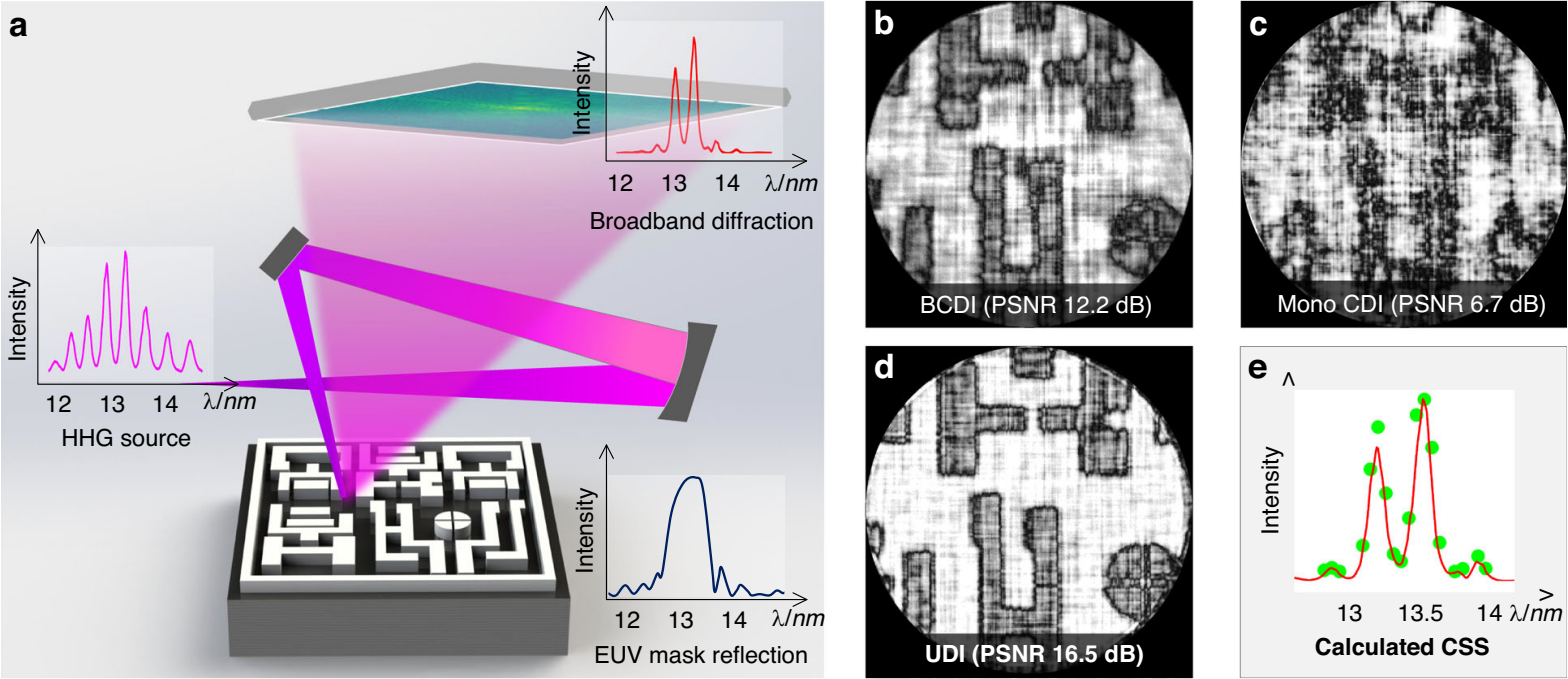
Broadband diffractive imaging with a spectrally chromatic EUV mask. **a** Schematic setup for a BCDI application with a chromatic EUV mask. **b**–**d** show the reconstructed images after 600 iterations of CDI using three different approaches: direct reconstruction from the recorded broadband diffraction pattern, mono CDI with a pre-knowledge of the broadband HHG source, and the proposed UDI, respectively. **e** The calculated CSS with only 18 sparse points of the spectrum

Figure 5 depicts a numerical simulation of BCDI for a spectrally dispersive EUV mask using broadband HHG source with 22% FWHM bandwidth spanning from 12 nm to 15 nm (see the magenta curves in Fig. 5a). The EUV mask’s multilayer structure functions as a bandpass spectral filter, selectively reflecting the spectrum centered at 13.5 nm and absorbing the remaining wavelengths^46^ (as detailed in Supplementary S7). This behavior is depicted by the EUV mask reflection curves in Fig. 5a. As a result, the spectrum of the HHG source undergoes modulation, allowing only two HHG harmonics to reflect from the EUV mask. This results in a spectral bandpass radiation (see the red curves in Fig. 5a). The corresponding BCDI result confirms the phenomenon that a successful convergence of CDI is achieved for the broadband diffraction pattern reflected from the EUV mask (Fig. 5b). Contrastingly, the mono CDI, dependent on *prior* knowledge of the broadband HHG source spectrum, fails to converge (Fig. 5c). This failure is attributed to the modulation of the incident light spectrum by the dispersive EUV mask. In comparison, our proposed UDI method achieves the best reconstruction (Fig. 5d), simultaneously recovers the probe spectrum and the EUV mask’s dispersiveness with only 18 sparse spectral components from the recovered CSS (Fig. 5e). The resulting recovered image exhibits a high PSNR^47^ of better than 16.5 dB.

It should be emphasized that the UDI outperforms the existing mono CDI for two main reasons. Firstly, UDI accurately recovers the spectral information of the imaging system. In contrast, mono CDI relies heavily on precise *prior* spectral measurements. Due to the detector’s spectral nonlinearity or the sample’s spectral dispersion, there is a significant deviation between the spectrum measurement of the light source and the spectral features in the captured diffraction image, preventing accurate spectral characterization. Besides, UDI also offers comprehensive improvements in monochromatization, coherence enhancement, noise robustness, and wide-spectrum robustness. This results in superior ultra-broadband computational imaging outcomes.

## Discussion

### Accuracy of CSS calculation

**Fig. 6:**
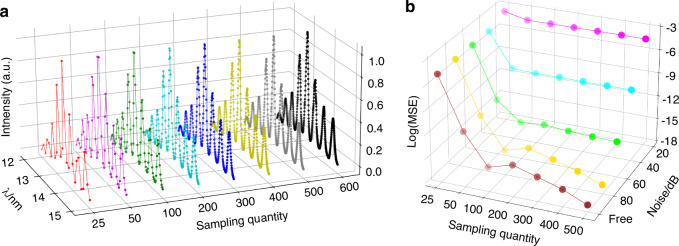
Broadband diffraction fitting error analysis. **a** Varying sampling intervals in HHG spectrum ranging from 12 nm to 15 nm. **b** The evolution of the broadband diffraction pattern fitting MSE with varying spectral sampling intervals in **a** and varying noise levels

We first performed a numerical investigation to evaluate how CSS affects the accuracy of broadband diffraction pattern fitting. We chose a broadband HHG source with a bandwidth of 22% FWHM as the illumination source in numerical calculation. Our analysis involved measuring the fitting error of a broadband diffraction pattern while varying the spectral sampling channels between 25 to 600 and the detector noise levels ranging from 20 dB to noisefree, as detailed in Fig. 6. The detector noise is a mixture with Gaussian noise and Poisson noise, following the detector noise model established in our previous work^48^.To assess the performance of the fitting, we used the mean squared error (MSE) as our evaluation function. Figure 6b demonstrates that the MSE decreases rapidly as the number of spectral samples increases, and stabilizes once the number of samples surpasses 100. Crucially, this trend of change is consistent across varying levels of noise. It reveals that a broadband diffraction pattern can be accurately decomposed into a sum of sparse, discrete channels of monochromatic diffraction patterns present in the source spectrum. This sparse sampled profile of spectrum represents the primary components of the full spectrum, which can be solved by the proposed UDI method.

**Fig. 7:**
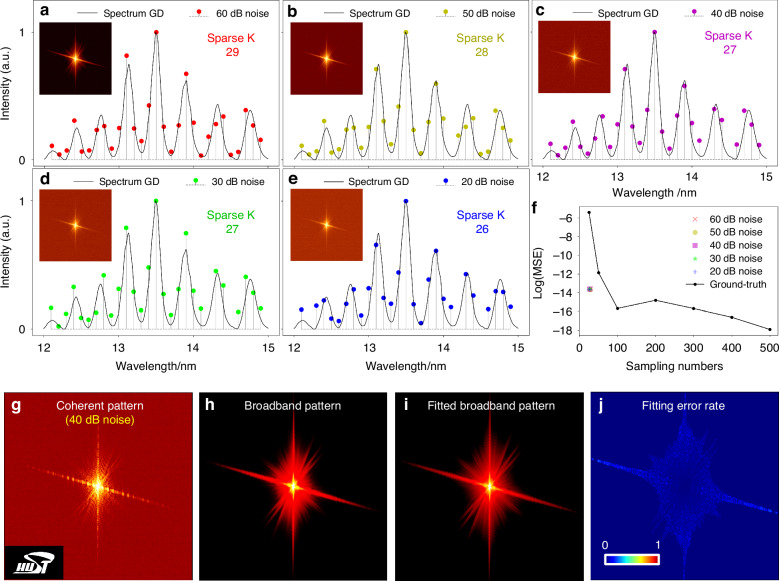
CSS accuracy analysis. **a**–**e** show the calculated CSS from the noised broadband patterns with varying detector noises ranging from 60 dB to 20 dB with 10 dB interval, respectively. **f** compares the MSE between the fitted broadband patterns generated from the calculated CSSs in **a**–**e** with the ground-truth spectrum, while varying the spectral sampling numbers. **g** A presentive shot of coherent diffraction pattern at 13.5 nm with 40 dB detector noise. **h**. The broadband diffraction pattern generated from the broadband HHG source in ground-truth with 600 discrete sampling points (black scatters in Fig. 6a), while the corresponding fitted broadband pattern from the calculated CSS under a noisy condition in **e** is presented in **i**, and its corresponding fitting error is displayed in **j**

We characterized the performances of CSS calculation with broadband diffractions under varying levels of noise. The “HSUT” logo (inside Fig. 7g) was used to generate ideal coherent diffraction data at 13.5 nm. Subsequently, a corresponding broadband pattern was obtained by linearly superimposing 600 discrete channels of monochromatic diffraction patterns in the HHG source spectrum. To replicate real-world scenarios, the diffraction datasets were synchronized with a 16-bit camera and added with varying levels of mixed detector noises.

Images inside Fig. 7a–e depict the broadband patterns with varying levels of noise, and the corresponding calculated CSSs are plotted simultaneously. Seeing that the calculated CSSs exhibit a strong correspondence with the spectrum within fewer than 30 spectral channels, even in the presence of substantial noise. We employed the sparsely calculated CSSs for fitting the broadband patterns, and subsequently analyzed the fitting MSEs and fitting error rates, as depicted in Figs. 7i, f, and j, respectively. The fitting MSEs consistently demonstrate minimal values across different noise levels, and the fitting error rate remains below 10%, displaying uniformity (Fig. 7j). These findings indicate that the calculated CSS effectively aligns with the primary components of the full-spectrum radiation, maintaining high compressed sparsity and robustness against noise.

### Broadband diffraction monochromatization in CSM

**Fig. 8:**
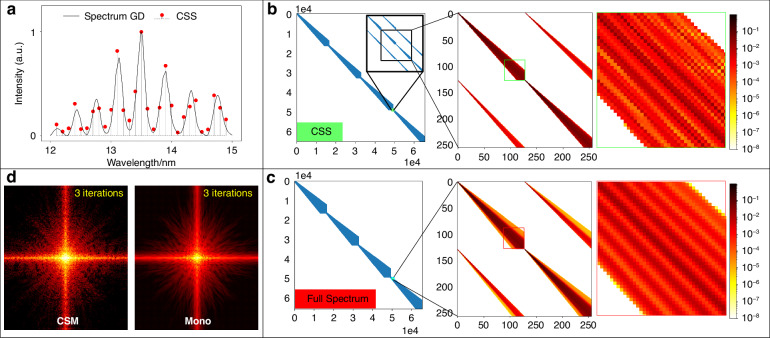
Broadband diffraction monochromatization. **a** The calculated CSS from the broadband pattern with 60 dB detector noise. **b** A 2D matrix C calculated from the CSS with 29 sparse spectral points in **a**. **c** Similar with **b** but calculated from the full spectrum with 600 sampled spectral channels for comparison. **d** The recovered monochromatized patterns after 3 iterations of CSM and mono, respectively

We utilized the recovered CSS with a bandwidth of 22% and 29 sparse spectral channels to create the sparse matrix C (Fig. 7a). Known that C is a sparse diagonal matrix with a sparsity of 0.03%. There are four identical sets of data distributed along the diagonal of matrix C. This is due to that the scaling of the spectral PSF is the same for all four quadrants. Figure 8b, c show the matrix C created from the CSS and the spectrum measurement, respectively for comparison. The matrix C created from the CSS has a distribution similar to that from the spectrum measurement, with only small localized differences where the sparse C is slightly non-uniform due to spectral leakage in CSS. However, these artifacts are not dominant in monochromatization due to the superiority of the proposed UDI method.

The generation of the matrix C from the CSS allows us to recover the optimal monochromatization from a broadband measurement. Figure 8d compares the monochromatized diffraction patterns between the mono method^34^ and the CSM process in UDI after 3 iterations. As a comparison, the CSM exhibits super-fast and smooth semi-convergence in monochromatization, generally well-retrieved monochromatization with enhanced coherence within the initial several iterations, whereas the mono has not yet found the direction and still exhibits similarities to the broadband diffraction with a mixture of decoherence. Additionally, a video sequence (Visualization 1) is also presented to show the evolution of CSM vs. mono. To further verify the superior noise-robustness of the proposed UDI method, an exhaustive comparison of BCDI reconstructions was conducted between UDI and mono CDI under diverse noise conditions. This detailed comparison is detailed in Supplementary S8.

### Outlook

**Fig. 9:**
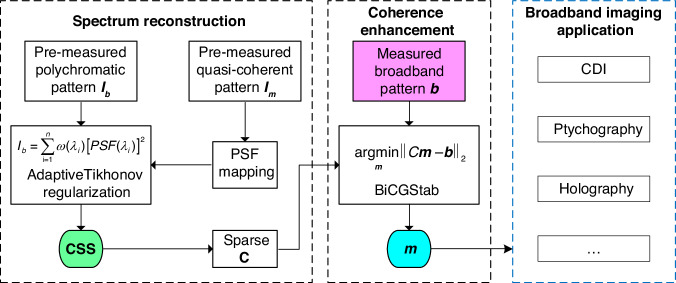
UDI workflow. The sparse matrix C is constructed as a pre-calculation process in the broadband CDI application, as indicated by the red dashed box. Subsequently, the measured broadband pattern ***b*** is monochromatized using the pre-calculated matrix C, resulting in the monochromatic pattern ***m***, as depicted in the blue dashed box. Notably, this monochromatization process does not require any *prior* knowledge of the spectrum

We have introduced a powerful UDI method for ultra-broadband diffractive imaging. Our research comprehensively addresses the key challenges of current state-of-art BCDI. By employing UDI, we successfully achieve a significant enhancement in coherence of ultra-broadband diffraction patterns. We provide a detailed explanation of the theory and design process for our UDI method in broadband diffractive imaging, which has been experimentally verified. It presents a natural sort of superiorities:

Firstly, UDI represents an advancement in ultra-broadband diffractive imaging with an unknown probe spectrum, while simultaneously recovering the spectrum information of the diffracted radiation. UDI overcomes limitations posed by constraints on spectrally non-dispersive specimens across a wide spectrum. It is inherently applicable across a broad wavelength range and eliminates the need for *prior* spectral knowledge, particularly crucial for applications in EUV and soft X-ray ranges where the absorption edge effects of materials are more pronounced. Figure 9.

Secondly, the UDI achieves coherence-enhanced, superfast-solving, and noise-robust monochromatization under ultra-wide spectral illumination. It efficiently utilizes the entire flux from a broadband source and results in a significant reduction in data acquisition time. This makes UDI highly beneficial for ultra-broadband imaging, offering a nearly fourfold improvement in bandwidth compared to existing mono CDI benchmark. The monochromatization in UDI operates with high efficiency, achieving optimal results within the initial iteration, which is 30 times faster than the state-of-the-art CGLS method^34,39^. Computational costs are detailed in Supplementary S4. The advancements are exclusively achieved within an ultra-streamlined BCDI architecture, offering substantial advantages for in-line broadband imaging.

Nevertheless, certain critical matters still require clarification and warrant further research. Despite the potent ultra-broadband imaging capabilities exhibited by the UDI when handling unknown probe spectrum, it is crucial to acknowledge that UDI relies on the Fresnel diffraction approximation. Consequently, its applicability may be constrained in scenarios involving multi-layer or multi-scattering samples, potentially impeding its capacity to fully leverage the entire performance in such intricate situations. Additionally, the optimization algorithm for monochromatization and the spectral quantum efficiency of the detector may also limit further spectral bandwidth extension in UDI. Our UDI experiments demonstrate ultra-broadband diffractive imaging with a relative spectral bandwidth exceeding 40% FWHM. This bandwidth is currently limited by the detector’s quantum efficiency, not by the UDI algorithm itself.

## Materials and methods

### CSS calculation

As described in Supplementary S1, Eq. (2) can be treated as an ill-posed multi-variable linear regression problem, which can be solved by Tikhonov regularization, to prevent overfitting and suppress the noise signals during reconstruction^37–39^. The least square of sum of squared residuals with a regularization item is minimized as

3 $${I}_{b}=\mathop{\sum }\limits_{{\rm{i}}=1}^{n}\omega ({\lambda }_{i}){[PSF({\lambda }_{i})]}^{2}\mathop{\Rightarrow }\limits^{Simplicity}\hat{{\boldsymbol{\omega }}}=\mathop{\rm{argmin}}\limits_{\omega }{\Vert {\boldsymbol{A}}{\boldsymbol{\omega }}-{\boldsymbol{b}}\Vert }_{2}^{2}+{\varGamma }^{2}{\Vert {\boldsymbol{\omega }}\Vert }_{2}^{2},\,\varGamma \,>\, 0$$

where ***A*** is a given M*N*n matrix with elements of each column of a flattened *PSF*(*λ*_*i*_) matrix in 1D array corresponding to the *i*-th slice of spectrum and ***b*** is a vector of a broadband diffraction flattened in 1D array, ***ω*** is the vector of unknown spectrum coefficients for the function. Γ is the regularization coefficient that controls the weight given to minimization of the side constraint relative to minimization of the residual norm. ||.||_2_ is the *l*_2_ norm. Note that the efficiency of these estimates depends on appropriately choice of the regularization coefficient $\Gamma$, which should be carefully selected to balance the results of robustness and resolution. In this work, we employ a generalized Cross-Validation statistic to make the balanced choice of Γ adaptively^49^:

4 $$\hat{\varGamma }=\mathop{\rm{argmin}}\limits_{\Gamma }\frac{{\Vert {\boldsymbol{A}}\hat{{\boldsymbol{\omega }}}-{\boldsymbol{b}}\Vert }_{2}^{2}}{{[n-Tr({\boldsymbol{A}}{({{\boldsymbol{A}}}^{T}{\boldsymbol{A}}+{\Gamma }^{2}I)}^{-1}{{\boldsymbol{A}}}^{T})]}^{2}}$$

where *I* is the identity matrix and the operator *Tr* sums elements on the main diagonal of a matrix. As a result, we can have the CSS estimates $\hat{{\boldsymbol{\omega }}}$ from solving Eq. (4)

5 $$\hat{{\boldsymbol{\omega }}}={({{\boldsymbol{A}}}^{T}{\boldsymbol{A}}{\boldsymbol{+}}{\Gamma }^{T}\Gamma )}^{-1}{{\boldsymbol{A}}}^{T}{\boldsymbol{b}}$$

The algorithm of the CSS calculation is described in detail in the supplementary information in ref. ^36^.

### Broadband monochromatization

As described in Supplementary S2, C is a sparse, symmetric, and positive definite matrix. The sparsity and positive definiteness of matrix C make Eq. (3) particularly well-suited for iterative solutions using BiCGStab. This algorithm performs as an implementation of an orthogonal projection technique onto the Krylov subspace. It involves minimizing the least squares problem to achieve the desired monochromatization. Implicitly, BiCGStab solves not only the original system $C{\boldsymbol{m}}={\boldsymbol{b}}$ but also a dual linear system ${C}^{T}{{\boldsymbol{m}}}^{{\boldsymbol{* }}}={{\boldsymbol{b}}}^{{\boldsymbol{* }}}$ with ${C}^{T}$.

6 $$\begin{array}{c}\begin{array}{cc}{\hat{\boldsymbol{m}}}=\mathop{\rm{argmin}}\limits_{{\boldsymbol{m}}}{\Vert C{\boldsymbol{m}}-{\boldsymbol{b}}\Vert }_{2} & {subject}\,{to}\,{\boldsymbol{m}}\in {{\mathcal{K}}}_{{k}}({C}^{T}C,{C}^{T}{\boldsymbol{b}})\end{array}\\ \begin{array}{cc}{{\hat{\boldsymbol{m}}}}^{\ast }=\mathop{\rm{argmin}}\limits_{{{\boldsymbol{m}}}^{\ast }}{\Vert {C}^{T}{{\boldsymbol{m}}}^{\ast }-{{\boldsymbol{b}}}^{\ast }\Vert }_{2} & {subject}\,{to}\,{{\boldsymbol{m}}}^{\ast }\in { {\mathcal L} }_{{k}}(C{C}^{T},C{{\boldsymbol{b}}}^{\ast })\end{array}\end{array}$$

where ${{\mathcal{K}}}_{k}\perp {{\mathcal{L}}}_{k}$, denotes the Krylov space orthogonally

$$\begin{array}{c}{{\rm{K}}}_{{k}}({\rm{C}}^{T}{\rm{C}},{\rm{C}}^{T}{\boldsymbol{b}}){\rm{f}}={\rm{span}}\{{\rm{C}}^{T}{\boldsymbol{b}},{\rm{C}}^{T}{\rm{C}}{\rm{C}}^{T}{\boldsymbol{b}},\ldots ,{({\rm{C}}^{T}{\rm{C}})}^{k-1}{\rm{C}}^{T}{\boldsymbol{b}}\}\\ {{\rm{L}}}_{{k}}({\rm{C}}{\rm{C}}^{T},{\rm{C}}{{\boldsymbol{b}}}^{\ast }){\rm{f}}={\rm{span}}\{{\rm{C}}{{\boldsymbol{b}}}^{\ast },{\rm{C}}{\rm{C}}^{T}{\rm{C}}{{\boldsymbol{b}}}^{\ast },\ldots ,{({\rm{C}}{\rm{C}}^{T})}^{k-1}{\rm{C}}{{\boldsymbol{b}}}^{\ast }\}\end{array}$$

The flowchart of BiCGStab algorithm is detailed in Supplementary S3.

### UDI workflow

**Step 1**: Pre-capture a shot of broadband pattern and a quasi-coherent pattern in-situ, respectively.

**Step 2**: Calculate the CSS from the measurements in **Step 1** via adaptive Tikhonov regularization.

**Step 3**: Calculate the sparse matrix C which contains the spectral information of CSS.

**Step 4**: Monochromatize the broadband patterns in broadband imaging experiments via BiCGStab along with the pre-calculated sparse matrix C in **Step 3**.

**Step 5**: Output the optimal diffraction pattern ***m*** with enhanced-coherence in broadband imaging applications.

### Supplementary information

Supplemental Information for Ultra-broadband Diffractive Imaging with Unknown Probe Spectrum

Visualization_1

## Data Availability

The data and codes that support the plots within this paper and other findings of this study are available from the corresponding author upon reasonable request. Source data are provided with this paper.

## References

[1] Chapman, H. N. & Nugent, K. A. Coherent lensless X-ray imaging. *Nat. Photonics* **4**, 833–839 (2010). 10.1038/nphoton.2010.240

[2] Miao, J. W. et al. Extending the methodology of X-ray crystallography to allow imaging of micrometre-sized non-crystalline specimens. *Nature* **400**, 342–344 (1999). 10.1038/22498

[3] Thibault, P. et al. High-resolution scanning X-ray diffraction microscopy. *Science* **321**, 379–382 (2008). 18635796 10.1126/science.1158573

[4] Gerchberg, R. W. & Saxton, W. O. Practical algorithm for the determination of phase from image and diffraction plane pictures. *Opt. (Stuttg.)* **35**, 237–246 (1972).

[5] Fienup, J. R. Reconstruction of an object from the modulus of its Fourier transform. *Opt. Lett.* **3**, 27–29 (1978). 19684685 10.1364/OL.3.000027

[6] Elser, V. Phase retrieval by iterated projections. *J. Optical Soc. Am. A* **20**, 40–55 (2003). 10.1364/JOSAA.20.000040 12542317

[7] Luke, D. R. Relaxed averaged alternating reflections for diffraction imaging. *Inverse Probl.* **21**, 37–50 (2005). 10.1088/0266-5611/21/1/004

[8] Eisebitt, S. et al. Lensless imaging of magnetic nanostructures by X-ray spectro-holography. *Nature* **432**, 885–888 (2004). 15602557 10.1038/nature03139

[9] Zhang, W. H. et al. Twin-image-free holography: a compressive sensing approach. *Phys. Rev. Lett.* **121**, 093902 (2018). 30230890 10.1103/PhysRevLett.121.093902

[10] Rodenburg, J. M. & Faulkner, H. M. L. A phase retrieval algorithm for shifting illumination. *Appl. Phys. Lett.* **85**, 4795–4797 (2004). 10.1063/1.1823034

[11] Maiden, A., Johnson, D. & Li, P. Further improvements to the ptychographical iterative engine. *Optica* **4**, 736–745 (2017). 10.1364/OPTICA.4.000736

[12] Sha, H. Z., Cui, J. Z. & Yu, R. Deep sub-angstrom resolution imaging by electron ptychography with misorientation correction. *Sci. Adv.* **8**, eabn2275 (2022). 35559675 10.1126/sciadv.abn2275 PMC9106290

[13] Jiang, S. W. et al. Resolution-enhanced parallel coded ptychography for high-throughput optical imaging. *ACS Photonics* **8**, 3261–3271 (2021). 10.1021/acsphotonics.1c01085

[14] Zheng, G. A., Horstmeyer, R. & Yang, C. Wide-field, high-resolution Fourier ptychographic microscopy. *Nat. Photonics* **7**, 739–745 (2013). 25243016 10.1038/nphoton.2013.187 PMC4169052

[15] Li, S. et al. Far-field synthetic aperture imaging via fourier ptychography with quasi-plane wave illumination. *Adv. Photonics Res.* **4**, 2300180 (2023). 10.1002/adpr.202300180

[16] Whitehead, L. W. et al. Diffractive imaging using partially coherent X rays. *Phys. Rev. Lett.* **103**, 243902 (2009). 20366201 10.1103/PhysRevLett.103.243902

[17] Gardner, D. F. et al. Subwavelength coherent imaging of periodic samples using a 13.5 nm tabletop high-harmonic light source. *Nat. Photonics* **11**, 259–263 (2017). 10.1038/nphoton.2017.33

[18] Baksh, P. D. et al. Quantitative and correlative extreme ultraviolet coherent imaging of mouse hippocampal neurons at high resolution. *Sci. Adv.* **6**, eaaz3025 (2020). 32494674 10.1126/sciadv.aaz3025 PMC7195139

[19] Ravasio, A. et al. Single-shot diffractive imaging with a table-top femtosecond soft x-ray laser-harmonics source. *Phys. Rev. Lett.* **103**, 028104 (2009). 19659250 10.1103/PhysRevLett.103.028104

[20] Fienup, J. R. Phase retrieval for undersampled broadband images. *J. Optical Soc. Am. A* **16**, 1831–1837 (1999). 10.1364/JOSAA.16.001831

[21] Thibault, P. & Menzel, A. Reconstructing state mixtures from diffraction measurements. *Nature* **494**, 68–71 (2013). 23389541 10.1038/nature11806

[22] Batey, D. J., Claus, D. & Rodenburg, J. M. Information multiplexing in ptychography. *Ultramicroscopy* **138**, 13–21 (2014). 24413077 10.1016/j.ultramic.2013.12.003

[23] Goldberger, D. et al. Spatiospectral characterization of ultrafast pulse-beams by multiplexed broadband ptychography. *Opt. Express* **29**, 32474–32490 (2021). 34615317 10.1364/OE.433752

[24] Loetgering, L. et al. Tailoring spatial entropy in extreme ultraviolet focused beams for multispectral ptychography. *Optica* **8**, 130–138 (2021). 10.1364/OPTICA.410007

[25] Noom, D. W. E. et al. High-speed multi-wavelength Fresnel diffraction imaging. *Opt. Express* **22**, 30504–30511 (2014). 25606996 10.1364/OE.22.030504

[26] Chen, B. et al. Multiple wavelength diffractive imaging. *Phys. Rev. A* **79**, 023809 (2009). 10.1103/PhysRevA.79.023809

[27] Yao, Y. et al. Broadband X-ray ptychography using multi-wavelength algorithm. *J. Synchrotron Radiat.* **28**, 309–317 (2021). 33399582 10.1107/S1600577520014708 PMC7842233

[28] Abbey, B. et al. Lensless imaging using broadband X-ray sources. *Nat. Photonics* **5**, 420–424 (2011). 10.1038/nphoton.2011.125

[29] Baksh, P. D. et al. Wide-field broadband extreme ultraviolet transmission ptychography using a high-harmonic source: publisher’s note. *Opt. Lett.* **41**, 3057 (2016). 27367100 10.1364/OL.41.003057

[30] Goldberger, D. et al. Single-pulse, reference-free, spatiospectral measurement of ultrashort pulse-beams. *Optica* **9**, 894–902 (2022). 10.1364/OPTICA.462586

[31] Rana, A. et al. Potential of attosecond coherent diffractive imaging. *Phys. Rev. Lett.* **125**, 086101 (2020). 32909811 10.1103/PhysRevLett.125.086101

[32] Jansen, G. S. M. et al. Diffractive shear interferometry for extreme ultraviolet high-resolution lensless imaging. *Opt. Express* **26**, 12479–12489 (2018). 29801285 10.1364/OE.26.012479

[33] De Beurs, A. C. C. et al. Extreme ultraviolet lensless imaging without object support through rotational diversity in diffractive shearing interferometry. *Opt. Express* **28**, 5257–5266 (2020). 32121750 10.1364/OE.380056

[34] Huijts, J. et al. Broadband coherent diffractive imaging. *Nat. Photonics* **14**, 618–622 (2020). 10.1038/s41566-020-0660-7

[35] Witte, S. et al. Lensless diffractive imaging with ultra-broadband table-top sources: from infrared to extreme-ultraviolet wavelengths. *Light Sci. Appl.* **3**, e163 (2014). 10.1038/lsa.2014.44

[36] Chen, C. C., Gu, H. G. & Liu, S. Y. Ultra-simplified diffraction-based computational spectrometer. *Light Sci. Appl.* **13**, 9 (2024). 38177112 10.1038/s41377-023-01355-4 PMC10766968

[37] Phillips, D. L. A technique for the numerical solution of certain integral equations of the first kind. *J. ACM* **9**, 84–97 (1962). 10.1145/321105.321114

[38] Doicu, A., Trautmann, T. & Schreier, F. Tikhonov regularization for nonlinear problems. In: *Numerical Regularization for Atmospheric Inverse Problems*. Springer Berlin Heidelberg: Berlin, Heidelberg, 163-220 (2010).

[39] Bell, B. Solutions of Ill-posed problems. by A. N. Tikhonov, V. Y. Arsenin. *Math. Comput.* **32**, 1320–1322 (1978). 10.2307/2006360

[40] Van Der Vorst, H. A. Bi-CGSTAB: a fast and smoothly converging variant of Bi-CG for the solution of nonsymmetric linear systems. *SIAM J. Sci. Stat. Comput.* **13**, 631–644 (1992). 10.1137/0913035

[41] Hansen, P. C. REGULARIZATION TOOLS: a matlab package for analysis and solution of discrete ill-posed problems. *Numer. Algorithms* **6**, 1–35 (1994). 10.1007/BF02149761

[42] Liu, R. F. et al. Broadband ptychographic imaging with an accurately sampled spectrum. *Phys. Rev. A* **107**, 033510 (2023). 10.1103/PhysRevA.107.033510

[43] Chen, B. et al. Diffraction imaging: the limits of partial coherence. *Phys. Rev. B* **86**, 235401 (2012). 10.1103/PhysRevB.86.235401

[44] Dilanian, R. A. et al. Diffractive imaging using a polychromatic high-harmonic generation soft-x-ray source. *J. Appl. Phys.* **106**, 023110 (2009). 10.1063/1.3176976

[45] Tanksalvala, M. et al. Nondestructive, high-resolution, chemically specific 3D nanostructure characterization using phase-sensitive EUV imaging reflectometry. *Sci. Adv.* **7**, eabd9667 (2021). 33571123 10.1126/sciadv.abd9667 PMC7840142

[46] Wood II, O.et al. Alternative materials for high numerical aperture extreme ultraviolet lithography mask stacks. Proceedings of SPIE 9422, Extreme Ultraviolet (EUV) Lithography VI. San Jose: SPIE, 94220I. 2015)

[47] Horé, A. & Ziou, D. Image quality metrics: PSNR vs. SSIM. Proceedings of 20th International Conference on Pattern Recognition. Istanbul: IEEE, 2366-2369. 2010)

[48] Chen, C. C., Gu, H. G. & Liu, S. Y. Noise-robust ptychography using dynamic sigmoid-remolding. *Opt. Laser Technol.* **172**, 110510 (2024). 10.1016/j.optlastec.2023.110510

[49] Craven, P. & Wahba, G. Smoothing noisy data with spline functions: estimating the correct degree of smoothing by the method of generalized cross-validation. *Numerische Mathematik* **31**, 377–403 (1978). 10.1007/BF01404567

