# Supplementary material for: Ultra-broadband diffractive imaging with unknown probe spectrum
Source: Light Sci Appl. 2024 Aug 26;13:213. doi: 10.1038/s41377-024-01581-4 (PMC11347606; doi:10.1038/s41377-024-01581-4)
Supplement: Supplementary file 1 — Supplemental Information for Ultra-broadband Diffractive Imaging with Unknown Probe Spectrum [file 41377_2024_1581_MOESM1_ESM.docx]

Supplemental Information for Ultra-Broadband Diffractive Imaging with Unknown Probe Spectrum

Chuangchuang Chen^a^, Honggang Gu^a,b,*^, Shiyuan Liu^a,b,*^

*^a^ State Key Laboratory of Intelligent Manufacturing Equipment and Technology, Huazhong University of Science and Technology, Wuhan, Hubei 430074, China*

*^b^ Optics Valley Laboratory, Wuhan, Hubei 430074, China*

*^*^ Corresponding authors:* [*hongganggu@hust.edu.cn*](mailto:hongganggu@hust.edu.cn) *(H. Gu);* [*shyliu@hust.edu.cn*](mailto:shyliu@hust.edu.cn) *(S. Liu)*

**S1 Broadband Fraunhofer diffraction approximation from PSF superposition**

Consider a monochromatic plane wave with a wavelength λ propagated from a microstructure couples the amplitude and phase of a diffraction field 𝜓*_λ_*(*x,* *y,* *z*) by traveling a distance of *z*, in the paraxial approximation, given by the Fraunhofer diffraction formula^1^:

, (S1)

where $\mathcal{F}$ denotes the 2D spatial Fourier transform of the sample *U*(*x′,* *y′, 0*) at *z* = 0, with *u* and *v* the spatial frequencies. In case of broadband radiation, the broadband diffracted field Փ can be written as:

$\Phi(\lambda)=\sqrt{\omega\left(\lambda\right)}\psi_{\lambda}(x,y,z)$, (S2)

where *ω*(*λ*) is the final corrected broadband radiation spectrum for the sample's spectral transmissivity function *T*(*λ*) and the detector's *QE*(*λ*), given as:

$\omega\left(\lambda\right)=S\left(\lambda\right)T\left(\lambda\right)QE\left(\lambda\right)$, (S3)

where the *S*(*λ*) denotes the initial spectrum radiation.

Since only the amplitude of diffraction is recorded by the detector, while the phase information is dropped, the detector integrates over time to produce the broadband diffraction pattern *I_b_*:

$I_{b}=\int\left|\Phi(t)\right|^{2}dt=\frac{1}{2\pi}\int\left|\Phi(\lambda)\right|^{2}d\lambda$, (S4)

with using Parseval’s theorem. By Eq. (S2) substituted into Eq. (S4), we have *I_b_*:

$I_{b}=\frac{1}{2\pi}\int\omega\left(\lambda\right)\left|\psi_{\lambda}\right|^{2}d\lambda$. (S5)

Known that a recorded monochromatic diffraction pattern *I_λ_* can be written as:

. (S6)

Seeing that the distribution of a Fraunhofer diffraction pattern depends only on the propagation distance z and wavelength λ in an identical way, showing a wavelength-dependent scaling factor *c*/*λz*, allowing us to map a coherent diffraction *I_λ_* at an arbitrary wavelength from a single diffraction shot *I_m_* at $\lambda_{m}$ by PSF propagation between different spectral components. Introducing the scaling factor *λ_i_*/*λ_m_*, the PSF mapping can be described as:

$PSF\left(\lambda_{i},x_{i},y_{i}\right)=\frac{c}{\lambda_{i}z}\begin{matrix} \frac{\lambda_{i}}{\lambda_{m}} & 0 & M\frac{\lambda_{m}-\lambda_{i}}{\lambda_{m}} \\ 0 & \frac{\lambda_{i}}{\lambda_{m}} & N\frac{\lambda_{m}-\lambda_{i}}{\lambda_{m}} \end{matrix}\left|\begin{matrix} x_{m} \\ y_{m} \\ 1 \end{matrix}\right|$, (S7)

where *x_i_, y_i_* denotes the coordinates of the diffraction field $\left| \psi_{i} \right|$ at a wavelength *λ_i_*, and M*,* N is the total number of pixels in the captured diffraction pattern. Seeing that the *PSF*(*λ_i_*) is an affine transformation from a reference diffraction filed $\sqrt{I_{m}}$ where *λ_i_*/*λ_m_* is the scaling factor to describe the PSF mapping and (M(*λ_m_ -λ_i_*)/*λ_m_,* N(*λ_m_ -λ_i_*)/*λ_m_*) is the translation factor to center the scaled diffraction orders.

Thus, combined with Eq. (S5~S7), the broadband diffraction pattern $I_{b}$ can be approximately rewritten as an integration of PSFs from the reference diffraction filed $\sqrt{I_{m}}$, weighted by the power spectrum *ω*(*λ*) over full spectral bandwidth of radiation:

$I_{\text{b}}=\int\omega(\lambda)\left[PSF(\lambda)\right]^{2}d\lambda$. (S8)

**S2 Sparse matrix C building in UDI**

As descripted in Eq. (S8), a measured broadband diffraction $I_{\boldsymbol{b}}$ can be thought of the integral of *ω*(*λ*)[*PSF*(*λ*)]^2^ over the wavelength range. For a given broadband diffraction ***b***, the retrieval of the monochromatic pattern ***m*** is reduced to a linear algebra problem, rewritten to a matrix form in simplicity:

$C^{T}b=C^{T}Cm$, (S9)

where ***m*** stands for the vector of the monochromatic pattern, ***b*** represents the broadband pattern, and the matrix C can be regarded as containing the spectrally dependent PSF in Eqs. (S7, S8). Here, we adopt a specific form of expression to calculate C in one dimension as outlined in ^2^, given by:

, (S9)

where

$$\begin{matrix} N=\left\{n:\left(j-1\right)\min\left\{\lambda_{c}/\lambda\right\}<n<j\left(\max\left\{\lambda_{c}/\left(\lambda+\lambda_{c}\right)\right\}\right)\right\}, \\ \Lambda=\left\{\lambda:\left(j-1\right)/n<\lambda/\lambda_{c}<j/\left(n-1\right)\right\}, \\ \lambda_{c}=\int_{\lambda} \hat{\omega}\left(\lambda\right)d\lambda/\sum\hat{\omega}\left(\lambda\right). \end{matrix}$$

Herein, *λ*, *n*, *j* are the indices that run over $\hat{\omega}\left( \lambda\right)$, ***m***, ***b*** respectively. The matrix C can be understood as the contribution of pixel *n* of ***m*** to pixel *j* of ***b*** is given by the part of pixel *n* that falls onto pixel *j* for the scaled pattern at wavelength *λ* by a scaled factor *λ*/ *λ_c_* times the corresponding CSS $\hat{\omega}\left( \lambda\right)$, summed over full spectrum $\Lambda$.

Ideally, the monochromatized ***m*** can be targeted to any wavelength within the broadband spectrum to retrieve the object’s response at any specific wavelength. However, in real-world conditions, we usually choose the spectrum's center of mass to minimize interpolation errors during the monochromatization calculation.

**S3 Enhancing monochromatization in UDI**

The primary difficulty with the discrete ill-posed problem in matrix function $\boldsymbol{b}=C\cdot\boldsymbol{m}$ is that it is essentially underdetermined due to the cluster of small singular values of the computed matrix C^3^. Hence, it is necessary to incorporate further information about the desired solution in order to stabilize the problem and to single out a useful and stable solution.

In our approach, we employ an enhanced version of the CG-S (Conjugate Gradients-Squared) method to address nonsymmetric linear systems. Specifically, we introduce a modified BiCGStab algorithm^4,5^ to effectively solve the monochromatization problem arising from broadband diffraction. BICGStab is performed with two additional constraints in this work: non-negativity of $\boldsymbol{m}_{k}$ (diffracted photon counts should not be negative) and a support constraint on the initial guess of $\boldsymbol{m}_{0}$ set to the measured broadband pattern **b**. These constraints help to prevent overfitting and further improve the regularizing power of the method. Thus, we obtain the following scheme for preconditioned Bi-CGSTAB:

$\boldsymbol{m}_{0}$is an initial guess;

$$r_{0}=\boldsymbol{b}-C\cdot\boldsymbol{m}_{\boldsymbol{0}}$$

$\bar{r}_{0}$is an arbitrary vector, such that

$\left( \bar{r}_{0}, r_{0} \right)\neq0,e.g., \bar{r}_{0}=r_{0}$;

$\rho_{0}=\alpha=\omega_{0}=1$;

$\upsilon_{0}=\alpha=p_{0}=0$;

for $i=1,2,3,\cdots,$

$\rho_{i}=\left( \bar{r}_{0}, r_{i} \right); \beta=\left( {\rho_{i}}/{\rho_{i-1}} \right)\left( \alpha/{\omega_{i-1}} \right);$

$p_{i}=r_{i-1}+ \beta\left( p_{i-1}-\omega_{i-1}\upsilon_{i-1} \right);$

Solve $y$ from $Ky=p_{i}$;

$\upsilon_{i}=Ay;$

$\alpha={\rho_{i}}/\left( \bar{r}_{0}, \upsilon_{i} \right);$

$s=r_{i-1}-\alpha\upsilon_{i};$

Solve $z$ from $Kz= s$;

$t=Az;$

$\omega_{i}=\left( K^{-1}t,K^{-1}s \right)/\left( K^{-1}t,K^{-1}t \right);$

$\boldsymbol{m}_{i}=\boldsymbol{m}_{i-1}+\alpha y+\omega_{i}z;$

$\boldsymbol{m}_{i}$ $\left[ \boldsymbol{m}_{i}<0 \right]=0;$

if $\boldsymbol{m}_{i}$ is accurate enough then quit;

$r_{i}=s-\omega_{i}t;$

End

**S4 UDI ptychography experimental set-up**

We describe the setup configuration that was employed for the broadband ptychography as shown in Fig. S1. In our experiment, we utilize a supercontinuum source (SC-Pro, YSL Photonics) with a repetition rate of 5 MHz to generate a broadband radiation. To select a specific range of wavelengths from the supercontinuum source, we employ a pair of spectral filters: a 600 nm short-pass filter, an 800nm short-pass filter, and a 450 nm long-pass filter. These filters allow us to extract the desired broadband spectra ranging from 450nm to 60 nm or from 450 nm to 800 nm for further measurements. A 90° flip narrowband filter with a center wavelength of 532 nm with a 3 nm FWHM (Full width at half maximum) (FL532-3, Thorlabs) is positioned on the optical path to generate a quasi-monochromatic radiation in situ. A 200 μm diameter pinhole (P200K, Thorlabs) is placed in front of the resolution target (R1L1S1N, Thorlabs) as a micro aperture to select the radiation to a spot size of approximately 200 μm. A CMOS detector (QHY268M, QHYCCD) is placed behind the sample at a distance of 30 mm to record the diffractions produced by the interaction of the radiation with the sample. The sample is mounted on an X-Y stage (M-L01K, PI). A sequence of broadband diffraction patterns is recorded as the sample is laterally scanned through the illuminated beam via a scanning probe with a step size of 30μm.

The Broadband Coherent Diffractive Imaging (BCDI) setup resembles that of broadband ptychography. The only minor distinction lies in the use of a 100 μm diameter pinhole (P100K, Thorlabs) in broadband CDI to generate a 100 μm diameter plane wave on the Siemens star target (R1L1S1N, Thorlabs) plane. Besides, the power of supercontinuum source is set to 80% of full power to meet with the detector’s dynamic range. The bandwidth of the spectrum extends from 475 nm to 605 nm.


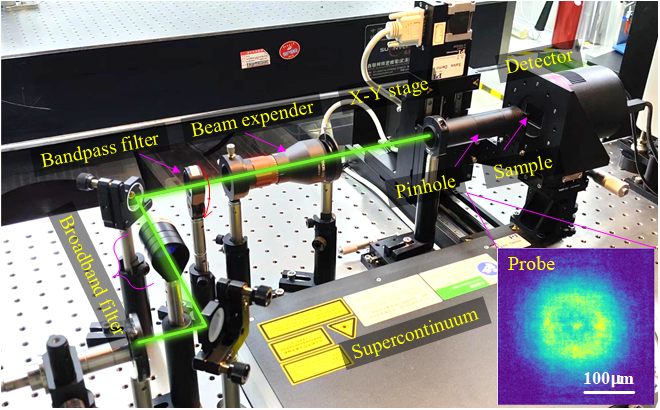


Fig. S1. Schematic of broadband ptychography setup. A broadband radiation was generated via a supercontinuum radiation passing through a set of broadband filters and expanded with a magnification of 5 by a beam expander, then directed onto the target through a 200 μm diameter pinhole and diffracted to the detector in the far field. A 90° flip narrowband filter at 532 nm with 3nm FWHM is placed on the optical path to generate a quasi-monochromatic radiation *in-situ*. The inside image depicts the reconstructed probe obtained through an ultra-broadband illumination with a 41% bandwidth using UDI ptychography.

In our experiment, we used an Intel i5-12400F CPU for the monochromatization calculations. As a result, our UDI method takes less than 150 ms to achieve optimal monochromatization, compared to over 5000 ms required by the mono CDI method. This highlights the significant advantage of our UDI method, particularly for in-line broadband imaging applications where efficiency and speed are critical.

**S5 Evaluation matric for broadband ptychography**

We monitored the evolution of the PSNRs between the reconstructed images and the ground-truth USAF target over the course of the ptychographic iterations, as plotted in Fig. S2(a). It can be seen that both broadband ptychography and UDI ptychography quickly established a sharp convergence within the first hundred iterations. However, the broadband ptychography subsequently got stuck in a local optimum, halting further improvement of the reconstructed image, while the UDI ptychography continued to converge at a high level. Eventually, after 600 iterations, the UDI ptychography achieved smooth convergence, resulting in a significantly improved reconstruction with a PSNR of 18 dB and clear resolution of all groups of features.

Note that in experimental data analysis, obtaining the true object complex function is not always feasible, making it challenging to calculate the PSNR for evaluation. Moreover, the conventional MSE metric used in diffraction analysis is primarily effective in coherent diffraction scenarios and not suitable for evaluation in cases involving broadband illumination. To overcome this, we propose an improved evolution function where we utilize the CSS and the recovered probe and object to fit a corresponding broadband diffraction dataset, enabling us to match with the original broadband measurement. This approach results in an improved error metric that better evaluates the quality of the reconstruction in broadband cases, given as

, (S10)

Where *J* denotes the total number of scanning positions in ptychography, *P*(*r*) and *O_j_*(*r*) denote the retrieved complex function of probe and object, respectively. Fig. S2(b) shows the plot of the corresponding error metric E in Eq. (S10) as a function of the iteration, demonstrating a similar trend to the PSNR evolution depicted in Fig. S2 (a). This observation confirms the effectiveness of the evaluation function in the context of broadband ptychography.

Fig. S2. Comparison of convergence evolution between broadband ptychography and CSM ptychography. **a** compares the evolution of the PSNR between the ground-truth USAF target pattern and the image recovery over 600 iterations of mPIE from the original broadband dataset (black) and the corresponding CSM dataset (green), respectively. **b** plots the evolution of the diffraction error E for the original broadband diffraction dataset (black), and the corresponding CSM dataset (green).

**S6 Monochromatization evolution under ultra-broadband illumination**


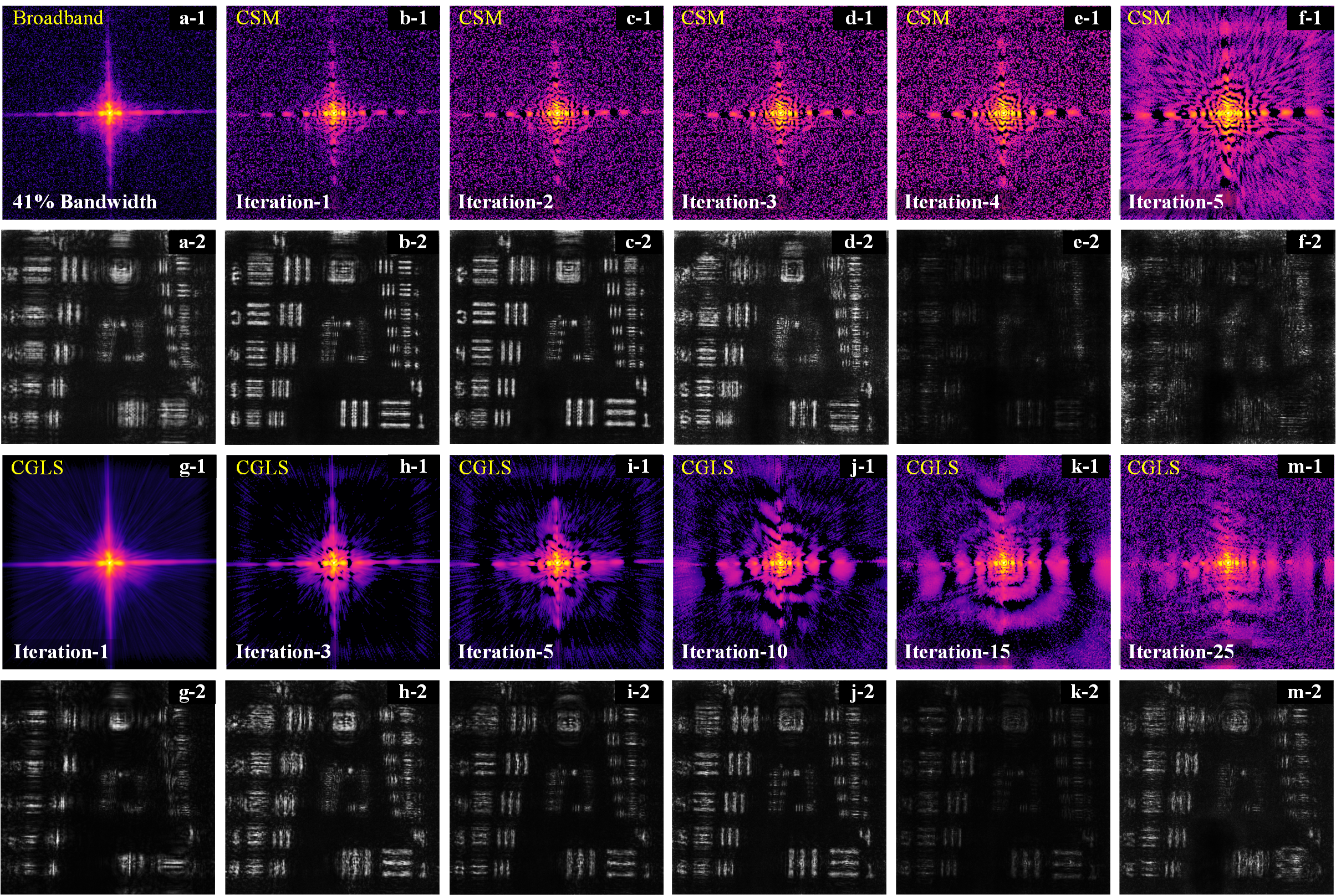


Fig. S3. Comparison of broadband diffraction monochromatization evolution between the CSM process in the proposed UDI method and the CGLS in mono CDI method at 41% bandwidth. **a-1** A frame of the ptychographic broadband diffractions at 41% bandwidth, and the corresponding ptychographic results are demonstrated in **a-2**. **b-1**~**f-1** show the monochromatization evolution within first 5 iterations of CSM, respectively, and the corresponding ptychography results are presented in **b-2**~**f-2**, respectively. **g-1**~**m-1** Monochromatization evolution at 1, 3, 5, 10, 15, 25 iterations of CGLS, respectively, **g-2**~**m-2** showcase the corresponding ptychography results, respectively.

We tracked the evolution of the monochromatization iterations using CSM process in the proposed UDI and CGLS in mono CDI method ^3^ from the ultra-broadband diffraction pattern with a 41% FWHM (Fig. 3**b** of the main text), as illustrated in Fig. S3. As evident from the results, even under the challenging conditions of an ultra-wide spectral bandwidth, our CSM approach efficiently calculates the monochromatized diffraction pattern within the first initial iteration (Fig. S3 **b**-1). A comparison with the original broadband pattern reveals a notable enhancement in the coherence of the CSM pattern, accompanied by a substantially high SNR. As the iterations continue (after 4 times of iterations), the CSM monochromatization exhibits overfitting, resulting in signal decoherence, and consequently, a reduction in the SNR. The corresponding ptychography results (Fig. S3 **b**2-**f**2) also confirm the characterization. In comparison, the monochromatization achieved by CGLS exhibits reduced sensitivity in coherence enhancement, leading to noise-induced blurring (Fig. S3 **g**1-**m**1). Consequently, this makes it ineffective for ultra-broadband ptychography (Fig. S3 **g**2-**m**2).

**S7 Reflective EUV mask structure**

In the EUV lithography process, an EUV mask reflects EUV light using multiple alternating layers of molybdenum and silicon^6^. The structure of a typical EUV mask is shown in Fig. S4. Unlike conventional photomasks, which block light with a single chromium layer on a quartz substrate, EUV light is strongly absorbed by most materials. Therefore, reflective optics, including the EUV mask structure, is carefully designed and applied to the EUV lithography tool. An EUV mask consists of 40-50 alternating silicon and molybdenum layers^7^. This multilayer structure reflects EUV light through Bragg diffraction. The reflectance of the best modern Mo/Si multilayers now approaches 70% in a narrow band of wavelengths near 13.5 nm. The TaN-based absorber stack is commonly used to fabricate the IC pattern on the EUV mask. The absorber layer must exhibit several characteristics, including high EUV absorption, stability under EUV radiation, and high etch selectivity.

**
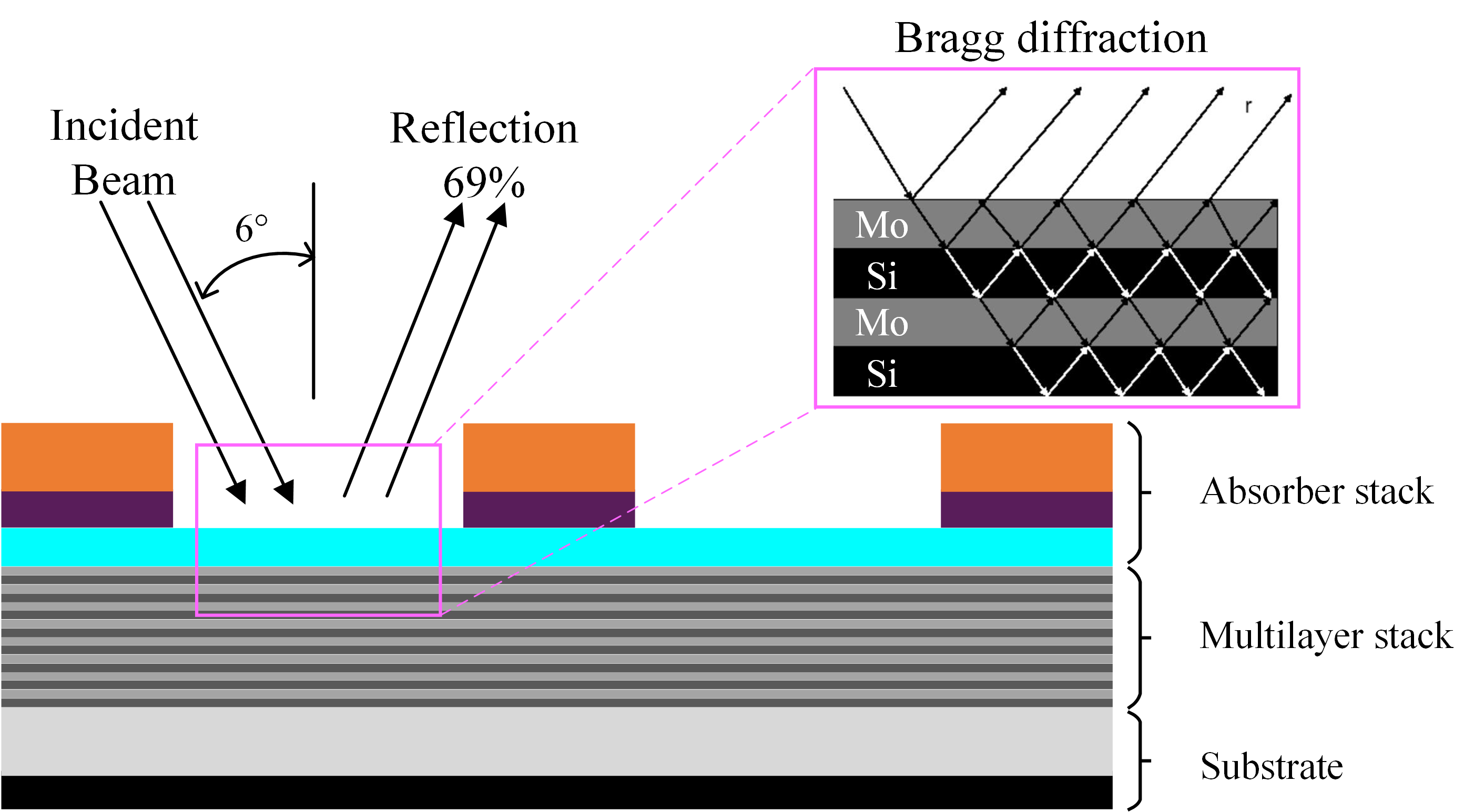
**

Fig. S4. The structure of an EUV reflective mask. The EUV mask blank is composed of 40-50 layers of Mo/Si multilayer to reflect the EUV light with a reflectance of nearly 70%. The TaN-based absorber stack is commonly used to fabricate the IC pattern on the EUV mask.

It should be mentioned that the Bragg diffraction occurs when radiation of a wavelength λ, comparable to the multilayer spacings, is scattered in a specular fashion and undergoes constructive interference. When the scattered waves are incident at a specific angle, they remain in phase and constructively interfere. That’s to say, the reflectance of the multilayer structure in the EUV mask depends on the incident angle and wavelength, with longer wavelengths reflecting more near normal incidence and shorter wavelengths reflecting more away from normal incidence^8^. It is observed that the EUV mask achieves its highest reflectance at a wavelength of 13.5 nm when the incidence angle is 6 degrees. Therefore, the multilayer EUV mask can be considered a high-performance bandpass filter reflector. This dispersive characteristics of the EUV mask modulates the incident light spectrum, leading to a notable discrepancy between the pre-measured spectrum of the light source and the spectral features observed in the captured broadband diffraction image. This discrepancy hinders precise spectral characterization in the existing mono CDI framework.

The 3D interaction of light with the EUV mask can introduce significant artifacts. The reflection and diffraction of EUV light, combined with all-reflective projection imaging, cause asymmetric shadowing, size bias, and telecentricity errors, leading to contrast variations. Thick absorbers deform the wavefront, creating aberration-like effects and variations in the best-focus position. Partial reflection from the absorber generates a weak secondary image that overlaps with the main image. The method described in this paper is applicable to wide-spectrum far-field scalar diffraction scenarios, which is independent of the near-field 3D effects of the EUV mask, such as multi-scattering and shadow effects.

**S8 Robustness of UDI against noise**

To further validate the enhanced noise-robustness of the proposed UDI method, we conducted a comprehensive comparison of BCDI reconstructions between UDI and mono CDI across various noise conditions. Fig. S5 illustrates the comparison between UDI and mono CDI under different levels of detector noise. The broadband diffraction pattern emanates from the HHG source with a 22% FWHM bandwidth, as depicted in Fig. 8**a** of the main text.

In the scenario of ideal diffraction data without noise, the reconstructed images from both mono CDI and the proposed UDI exhibit excellent recovery with high Peak Signal-to-Noise Ratio (PSNR), as illustrated in Fig. S5 **d1** and **f1**. Due to the diffraction aliasing in broadband radiation, the conventional BCDI fails to converge (Fig. S5 **b1**). Notably, it is essential to highlight that optimal monochromatization is achieved after 30 iterations in the case of mono CDI (Fig. S5 **c1**), whereas only a single initial iteration is required for UDI (Fig. S5 **e1**).

When subjected to a noisy diffraction dataset, in contrast to the noise-free scenarios portrayed in Fig. S5 **c1**, the monochromatization performance of mono CDI noticeably degrades. Specifically, for the diffraction dataset with 40 dB detector noise, the monochromatization process in mono proves highly susceptible to noise. The monochromatized pattern undergoes a significant increase in distortion and recovery errors, while the enhancement in coherence remains limited (Fig. S5 **c2**). This effect becomes even more pronounced as the detector noise is increased to 30 dB (Fig. S5 **c3**).


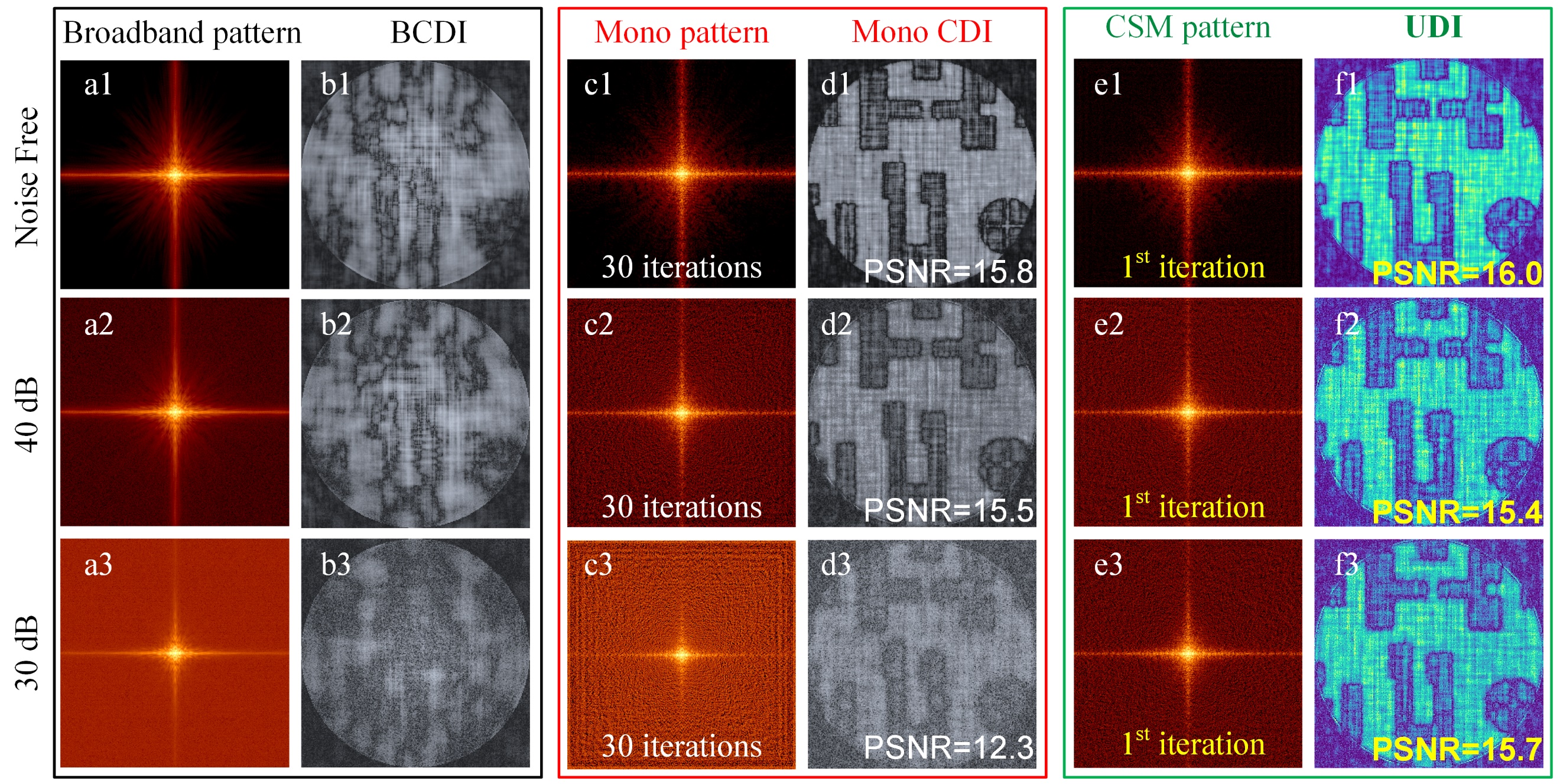


Fig. S5. **UDI under varying noise conditions. a1**-**a3**: Broadband patterns diffracted from the broadband HHG source with a 22% FWHM bandwidth under varying noise conditions: Noise free, 40 dB detector noise, and 30 dB detector noise, respectively. **b1**-**b3** depict the images reconstructed after 500 iterations of RAAR algorithm from the broadband diffraction datasets demonstrated in **a1**-**a3**, respectively. **c1**-**c3**: Similar with **a1**-**a3**, but monochromatized after 30 iterations of the mono CDI method^2^. **d1**-**d3**: Similar with **b1**-**b3**, but from the monochromatized diffraction datasets demonstrated in **c1**-**c3**. **e1**-**e3**: Similar with **c1**-**c3**, but monochromatized after only a single initial iteration of the proposed CSM method. **f1-f3**: The UDI reconstructions, which is similar with **d1-d3**, but from the monochromatized diffraction datasets demonstrated in **e1**-**e3**.

Contrastingly, the proposed UDI method consistently outperforms in achieving improved monochromatization, even in the presence of substantial noise. Notably, the UDI attains optimized monochromatization with significant coherence enhancement as early as the first initial iteration, even when confronted with challenging conditions such as a broadband diffraction pattern with a 22% FWHM bandwidth at 15 dB heavy detector noise (Fig. S5 **e**3).

The superior robustness of CSM is further substantiated in UDI. In comparison to mono CDI under noisy diffraction datasets, the reconstructed images from UDI consistently exhibit high levels of recovery, characterized by superior resolution and contrast, achieving a PSNR exceeding 15 dB (Fig. S5 **f2**, **f3**). In contrast, the result from mono CDI experiences a significant decrease, reaching only 12 dB (Fig. S5 **d2**, **d3**).

References

1 Ioseph W. Goodman. *Introduction to Fourier Optics*. 4th ed. Roberts and Company Publishers, 2005.

2 Huijts J, Fernandez S, Gauthier D, Kholodtsova M, Maghraoui A, Medjoubi K *et al.* Broadband coherent diffractive imaging. *Nat Photonics* 2020; **14**: 618–622.

3 Hansen P.C. REGULARIZATION TOOLS: A Matlab package for analysis and solution of discrete ill-posed problems. *Numer Algorithms* 1994; **6**: 1–35.

4 Fokkema DR, Sleijpen GLG, Van der Vorst HA. Generalized conjugate gradient squared. *J Comput Appl Math* 1996; **71**: 125–146.

5 van der Vorst HA. Bi-CGSTAB: A Fast and Smoothly Converging Variant of Bi-CG for the Solution of Nonsymmetric Linear Systems. *SIAM J Sci Stat Comput* 1992; **13**: 631–644.

6 Erdmann A, Xu D, Evanschitzky P, Philipsen V, Luong V, Hendrickx E. Characterization and mitigation of 3D mask effects in extreme ultraviolet lithography. *Adv Opt Technol* 2017; **6**: 187–201.

7 Wood O, Raghunathan S, Mangat P, Philipsen V, Luong V, Kearney P *et al.* Alternative materials for high numerical aperture extreme ultraviolet lithography mask stacks. In: Wood OR, Panning EM (eds). *Proc. SPIE*. 2015, p 94220I.

8 Philipsen V, Hendrickx E, Jonckheere R, Davydova N, Fliervoet T, Neumann JT. Actinic characterization and modeling of the EUV mask stack. In: Behringer UFW, Maurer W (eds). *Proc. of SPIE*. 2013, p 88860B.
